# Productivity model and analysis of influencing factors on horizontal fracture productivity in ultra-low permeability reservoir

**DOI:** 10.1371/journal.pone.0324463

**Published:** 2025-05-27

**Authors:** Xiaofeng Wang, Binchi Hou, Hailong Dang, Houjun Tang, Pengxing Cui, Tao Gao

**Affiliations:** 1 Research Institute of Shaanxi Yanchang Petroleum (Group) Co., Ltd., Xi’an, China; 2 Research Center of Exploration and Development Engineering for Extra Low Permeability Oil and Gas Fields in Shaanxi Province, Xi’an, China; 3 School of Energy Resources, China University of Geosciences, Beijing, China; Tribhuvan University, NEPAL

## Abstract

Ultra-low permeability reservoir is characterized by the complex seepage law, Darcy’s law is not applicable to which and it is difficult to predict vertical well horizontal fracture productivity effectively. In this study, according to the law of equivalent percolation resistance, vertical well fracturing horizontal fracture seepage field is divided into two areas by pseudo-well assumptions: the reservoir external seepage resistance generated outside pseudo-well, where seepage flow is radial flow. Reservoir inner seepage resistance generated in pseudo-well, where seepage flow is vertical linear unidirectional flow. Based on principle of hydroelectricity similarity, the inner with the external seepage resistance were combined in series, the vertical well horizontal fracture productivity prediction model was established, and the productivity influence factors were analyzed by simulation. The results demonstrated that horizontal permeability had a great influence on oil production, and vertical permeability had little influence on the production. The bigger starting pressure gradient existed the critical value, the oil wells production showed a marked decline with the value bigger.

## 1. Introduction

Since the 21st century, China’s increasing energy demands have necessitated the accelerated development of low-permeability oil reservoirs. The seepage law of low-permeability oil reservoirs is complex. As a result, most low-permeability oil reservoirs need to be developed by fracturing. The permeability of ultra-low permeability reservoirs is less than 1 × 10^-3^ µm^2^, making oil development more difficult. The productivity of fractured wells is an important factor to be considered in economic evaluation. Currently, a lot of research has been conducted both domestically and internationally on productivity prediction of fractured wells [1–8]. As for overseas research, Sohman [1] studied the law of productivity change of fractured wells with time. In China, Hu Jinghong et al. [5] developed a numerical model of horizontal fractured oil well productivity and analyzed the influence of permeability and other factors on productivity, while Wang Zhiping et al. [6] studied the productivity calculation of fractured horizontal wells. Wang Wendong et al. [7–12] proposed a multi-field coupled analytical theoretical model for shale oil fracturing. However, there are currently few studies on the productivity model of fractured horizontal fractures considering the starting pressure gradient or vertical permeability [13–22]. This study examines the effects of starting pressure gradient and vertical permeability on the productivity of fractured horizontal fractures in vertical wells. Moreover, a productivity calculation model of fractured horizontal fractures in vertical wells is developed using the equivalent seepage resistance method, and the influencing factors of productivity are simulated and analyzed. The development of this model provides a certain theoretical basis for the productivity prediction of fractured horizontal fractures in oil fields.

## 2. Productivity prediction model of fractured horizontal fractures in vertical wells

### 2.1. Model assumptions

(1) The reservoir is an infinite homogeneous low-permeability oil reservoir with closed top and bottom.
(2) The fluid in both the oil reservoir and fracture is single-phase fluid, slightly compressible, and the seepage is isothermal and stable, regardless of gravitational effects.
(3) The fractured fracture in vertical wells is horizontal, forming an elliptical plane in space, and the horizontal fracture is located at the vertical center of the reservoir.
(4) The influence of the starting pressure gradient and vertical permeability is considered.

### 2.2. Model building

**Fig 1:**
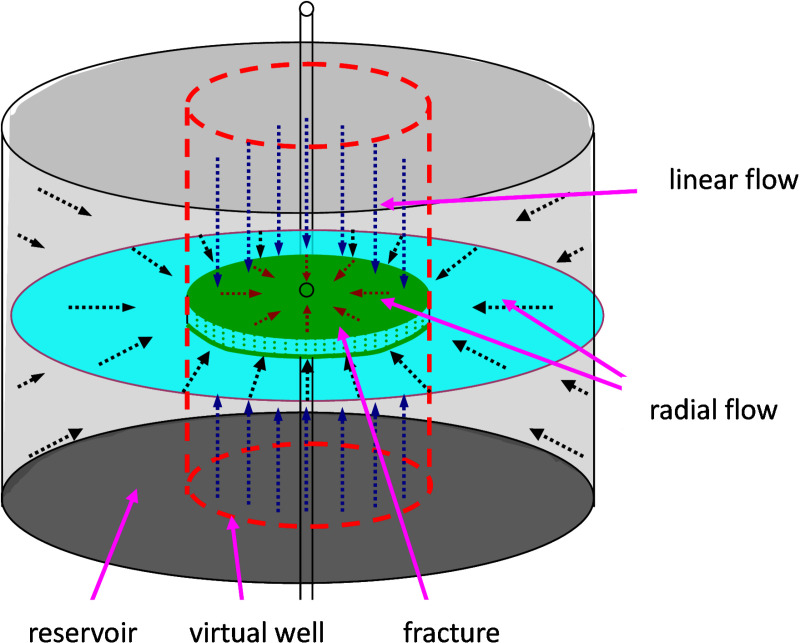
The model for the fractured vertical wells.

As shown in Fig 1, the horizontal fracture is an elliptical cylinder with a height equal to the width of the fracture in space. The cross-section of the horizontal fracture is extended to the top and bottom of the reservoir, forming an enlarged virtual well. The equivalent seepage resistance method is used to divide the flow area of the reservoir into two parts. The resistance of the flow area outside the virtual well is referred to as seepage external resistance, and the flow is radial flow, whereas the area inside the virtual well represents seepage internal resistance with vertical unidirectional linear flow.

#### 2.2.1. Seepage external resistance of the upper part of the reservoir outside virtual well.

According to the potential energy theory, the elliptical horizontal fracture also forms an elliptical drainage area in the horizontal direction. The major axis of the elliptical area is $a_{f}$ , and the minor axis is $b_{f}$, which is equivalent to a circular drainage area with a supply radius of $\left(a_{f}+b_{f}\right)/2$ and a production well diameter of virtual well radius.

Considering the influence of the starting pressure gradient on the seepage law of a low-permeability oil reservoir, the radial motion equation for the fluid can be expressed as follows:


$$v=\frac{k}{\mu }\left(\frac{dp}{dr}-G\right)$$ (1)

where G is the horizontal starting pressure gradient of reservoir, μ is the viscosity, k is the horizontal permeability of reservoir, v is the seepage velocity.

The boundary conditions of the external seepage field of the virtual well are expressed as follows:


$$r_{m}=\frac{a_{f}+b_{f}}{2},p=p_{m},r_{e}=\frac{a+b}{2},p=p_{e},h=\frac{H}{2}$$ (2)

where $a_{f}$ and $b_{f}$ are the major and minor axes of elliptical horizontal fractures, respectively. $r_{m}$ and $r_{e}$ are the radial distance from the center of the external seepage field wellbore to the supply edge and the equivalent radius of elliptical horizontal crack controlling the discharge area, respectively. $p_{e}$ and $p_{m}$ are the supply edge formation pressure and the pressure at the junction of external and internal flow fields, respectively. H is the thickness of the upper oil layer in the reservoir

Organize and integrate to obtain the following equation:


$$\int_{p_{m}}^{p_{e}}dp=\int_{r_{m}}^{r_{e}}\left(\frac{q\mu }{\pi kH}\frac{1}{r}+G\right)dr$$ (3)

Organize to the following equation:


$$q_{1}=\frac{\pi kH\left(p_{e}-p_{m}-G\left(r_{e}-r_{m}\right)\right)}{\mu \ln\frac{r_{e}}{r_{m}}}$$ (4)

The seepage resistance of the upper half of the reservoir outside the virtual well is expressed as follows:


$$R_{1}=\frac{\mu }{\pi kH}\ln\frac{r_{e}}{r_{m}}$$ (5)

#### 2.2.2. Seepage internal resistance of the upper part of the reservoir inside the virtual well.

Considering the influence of the starting pressure gradient on the seepage law of a low-permeability oil reservoir, the equation of vertical unidirectional linear flow of fluid within the virtual well can be expressed as follows:


$$v=\frac{k_{z}}{\mu }\left(\frac{dp}{dx}-G_{z}\right)$$ (6)

$$k_{z}=\frac{k_{v}h+k_{f}w_{f}}{h}$$ (7)

where $G_{z}$ is the vertical starting pressure gradient of reservoir, $k_{z}$ is the Equivalent permeability of internal seepage field, $k_{v}$ is the vertical permeability, $k_{f}$ is the horizontal fracture permeability, $w_{f}$ is the horizontal fracture width.

Therefore, The boundary conditions for the upper half of the seepage field inside the virtual well can be expressed as follows:


$$h=\frac{w_{f}}{2},p=p_{m};h=\frac{H}{2},p=p_{e};$$ (8)

Organize and integrate to obtain the following equation:


$$\int_{p_{w}}^{p_{m}}dp=\int_{\frac{w_{f}}{2}}^{\frac{H_{2}}{\;}}\left(\frac{q\mu }{\pi k_{z}r_{m}^{2}}+G_{z}\right)dx$$ (9)

Organize to the following equation:


$$q_{2}=\frac{\pi k_{z}r_{m}^{2}\left(p_{m}-p_{w}-G_{z}\left(\frac{H}{2}-\frac{w_{f}}{2}\right)\right)}{\mu \left(\frac{H}{2}-\frac{w_{f}}{2}\right)}$$ (10)

Thus, the internal resistance of seepage in the upper half of the reservoir inside the virtual well can be expressed as follows:


$$R_{2}=\frac{\mu \left(\frac{H}{2}-\frac{w_{f}}{2}\right)}{\pi k_{z}r_{m}^{2}}$$ (11)

#### 2.2.3. Productivity equation for fractured horizontal fractures in vertical wells.

According to the equivalent seepage resistance method, the seepage external resistance and the seepage internal resistance are in a series relationship. Therefore, the flow rate in the upper part of the reservoir can be obtained as follows:


$$q=\frac{\left(p_{e}-p_{w}-\left(G(r_{e}-r_{m})+G_{z}\left(\frac{H}{2}-\frac{w_{f}}{2}\right)\right)\right)}{R_{1}+R_{2}}$$ (12)

The total flow of the reservoir should be the sum of the flow rates from the upper and lower parts of the reservoir. Since the flow rates in the upper and lower parts of the reservoir are equal, the total flow of the reservoir can be expressed as:


$$Q=2q=\frac{2\left(p_{e}-p_{w}-\left(G(r_{e}-r_{m})+G_{z}\left(\frac{H}{2}-\frac{w_{f}}{2}\right)\right)\right)}{\frac{\mu }{\pi kH}\ln\frac{r_{e}}{r_{m}}+\frac{\mu (H-w_{f})}{2\pi k_{z}r_{m}^{2}}}$$ (13)

The daily production capacity of oil wells can be expressed as:


$$Q_{m}=\frac{2\rho_{o}\left(p_{e}-p_{w}-\left(G(r_{e}-r_{m})+G_{z}\left(\frac{H}{2}-\frac{w_{f}}{2}\right)\right)\right)}{B_{o}\left(\frac{\mu }{\pi kH}\ln\frac{r_{e}}{r_{m}}+\frac{\mu (H-w_{f})}{2\pi k_{z}r_{m}^{2}}\right)}$$ (14)

where $B_{o}$ is the oil volume factor, $\rho_{o}$ is the oil density.

## 3. Analysis of factors influencing the productivity of fractured horizontal fractures in vertical wells

### 3.1. Simulation parameters

The simulation parameters for the productivity model of fractured horizontal fracture in vertical wells are shown in Table 1:

**Table 1:** The values of the simulation parameters.

Factors	Value range	Factors	Value range
Formation pressure (MPa)	3~7	Bottom hole pressure (MPa)	0.5~1.5
Horizontal permeability (mD)	1~10	Oil viscosity (mPa·s)	5.1
vertical permeability (mD)	0.05~1	Oil volume factor	1.05
Horizontal fratures long half axis (m)	60~180	Oil density (g/cm3)	0.809
Horizontal fratures short half axis (m)	30~100	Reservoir thickness (m)	3~6

### 3.2. Influence of formation pressure and flowing pressure

**Fig 2:**
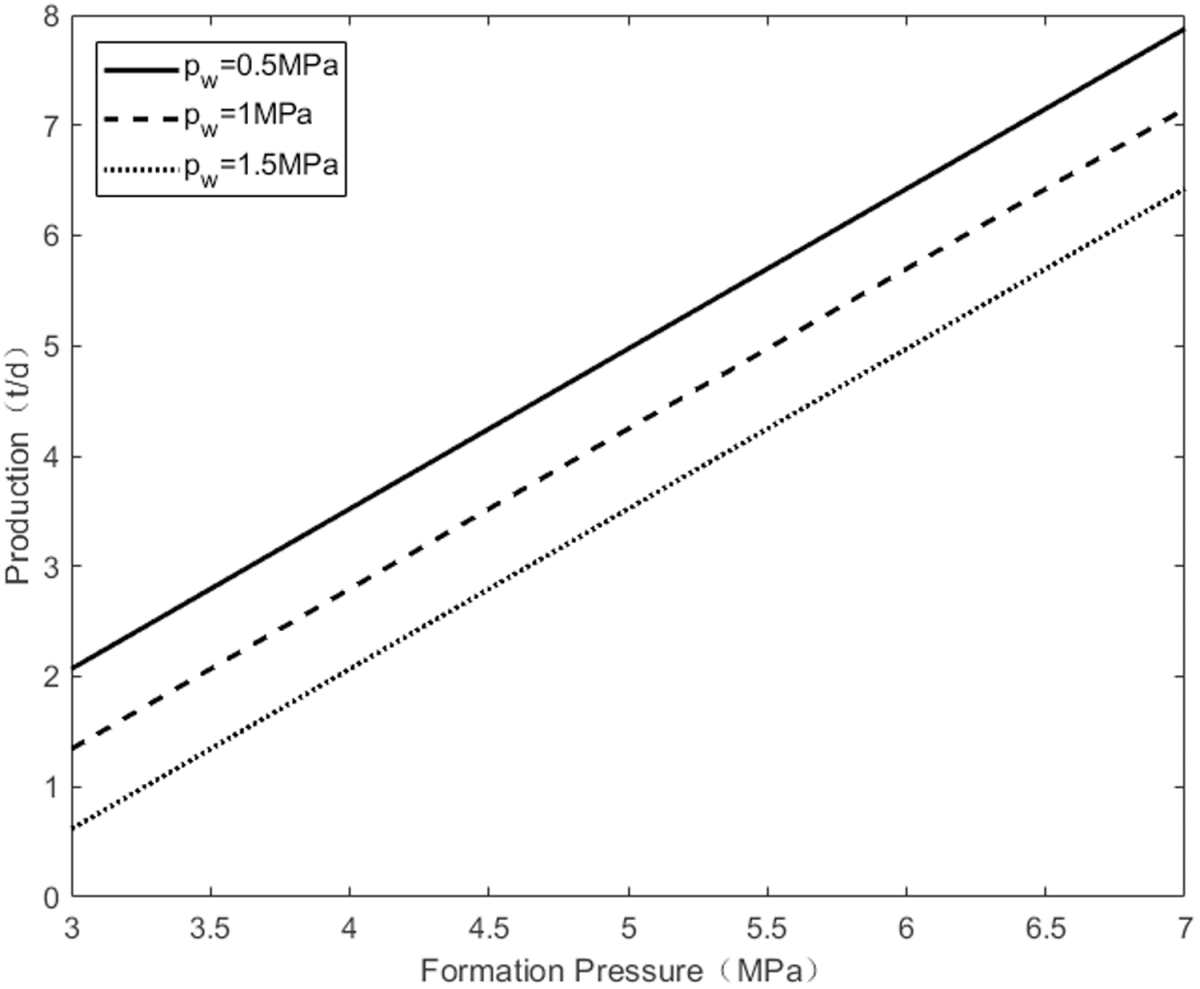
The change of pressure under different of bottom hole flowing pressure.

As shown in Fig 2, when the flowing bottomhole pressure is constant, an increase in formation pressure leads to a rise in the production differential pressure of the oil well, resulting in a gradual increase in daily oil production. There is a linear relationship between formation pressure and daily oil well production. Conversely, at the same formation pressure, an increase in flowing bottom pressure decreases the production differential pressure of oil wells, leading to a reduction in the daily production of oil wells.

### 3.3. Influence of reservoir permeability

**Fig 3:**
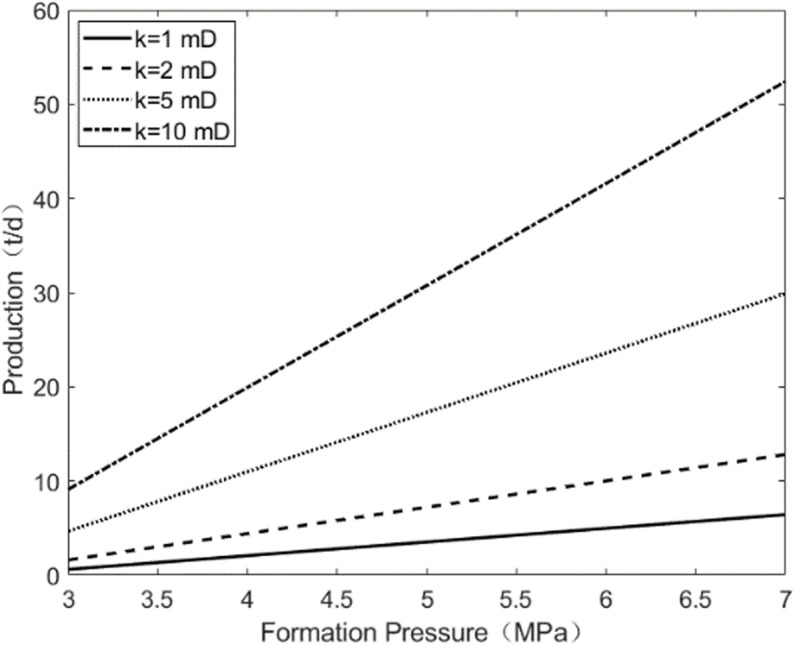
The change of pressure under the horizontal permeability.

**Fig 4:**
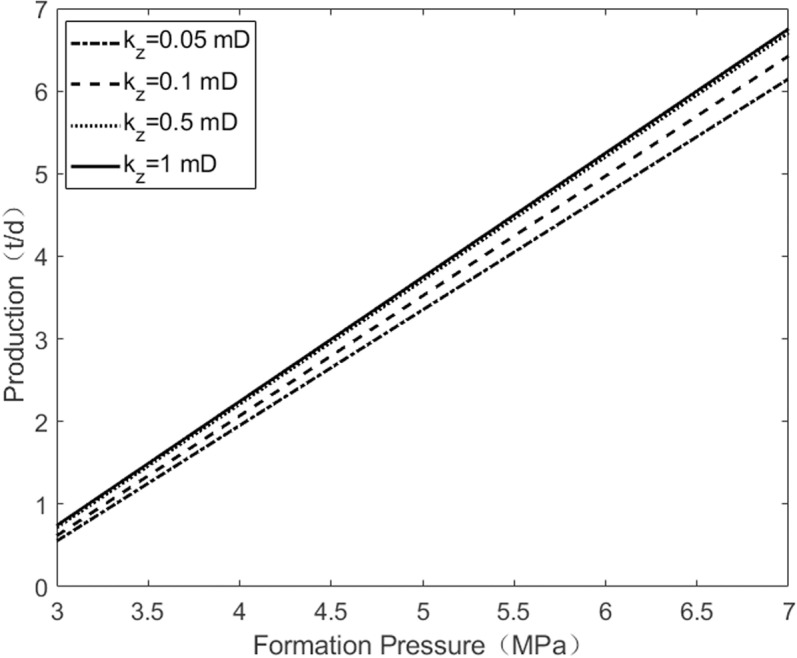
The change of pressure under the vertical permeability.

As shown in Fig 3, when the vertical permeability of the reservoir is constant, the horizontal permeability of the reservoir has a significant impact on the daily production of the oil well. As the horizontal permeability of the reservoir increases, the daily production of the oil well rises substantially. Additionally, with an increase in the formation pressure, the growth rate of the daily production of the oil well also increases. As shown in Fig 4, when the horizontal permeability of the reservoir is held constant, an increase in the vertical permeability of the reservoir weakens the vertical heterogeneity, leading to an increase in the daily production of the oil well. Comparing the results of Figs 3 and 4, horizontal permeability is the main factor affecting the daily production of oil wells, while vertical permeability has a negligible influence.

### 3.4. Influence of horizontal fracture parameters

**Fig 5:**
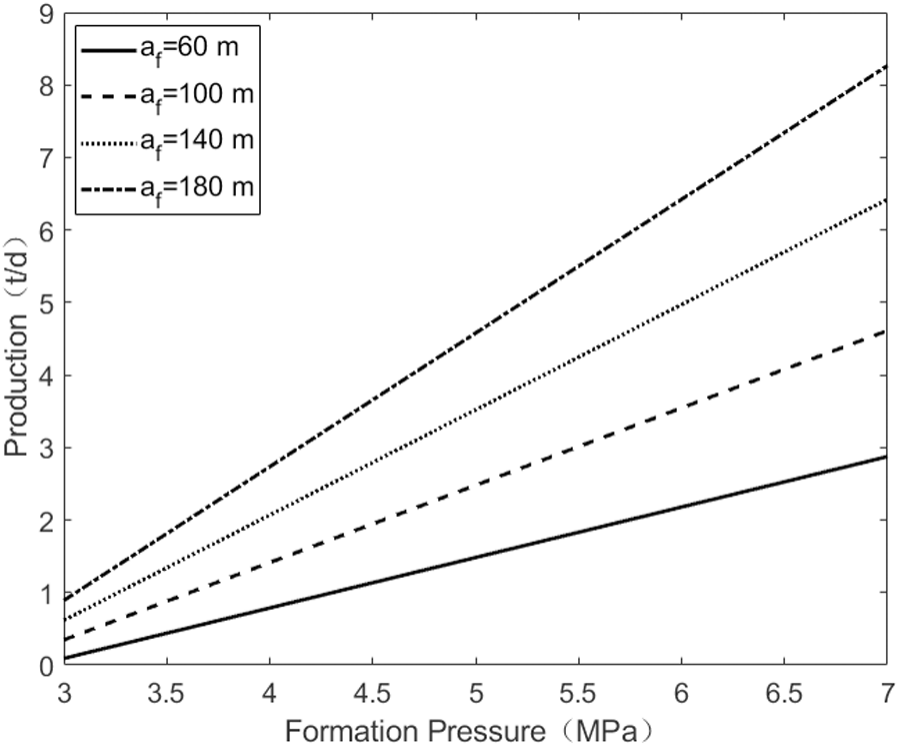
The change of pressure under the half of longer axes.

**Fig 6:**
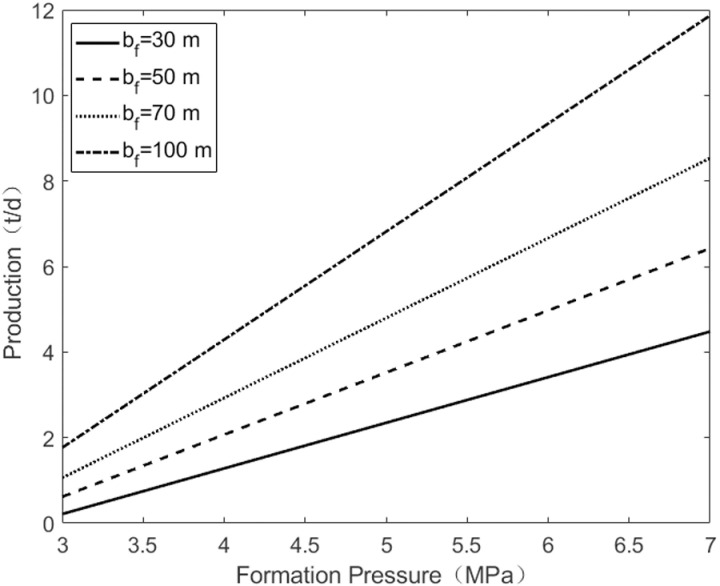
The change of pressure under the half of short axes.

As shown in Fig 5, when the semi-minor axis of the horizontal fracture remains unchanged, increasing the semi-major axis of the horizontal fracture from 60 m to 180 m expands the oil drainage area controlled by the fracture, resulting in an increase in oil well production from 2.9 t/d to 8.2 t/d. As shown in Fig 6, when the semi-major axis of the horizontal fracture remains constant, increasing the semi-minor axis of the horizontal fracture from 30 m to 100 m raises oil well production from 4.3 t/d to 12 t/d. This demonstrates that with the increase of the minor axis and major axis of horizontal fracture, the production of single oil wells increases, with the minor axis having a slightly greater impact on production than the major axis.

### 3.5. Influence of starting pressure gradient

**Fig 7:**
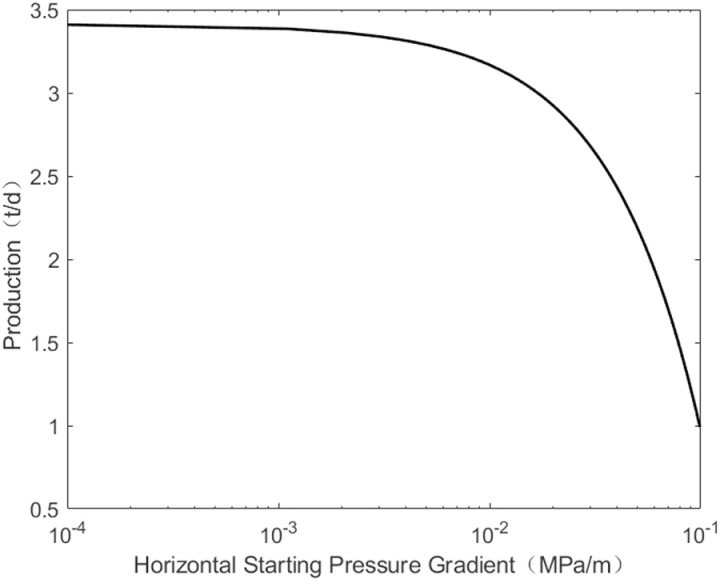
The change of pressure under horizontal of start-up pressure gradient.

**Fig 8:**
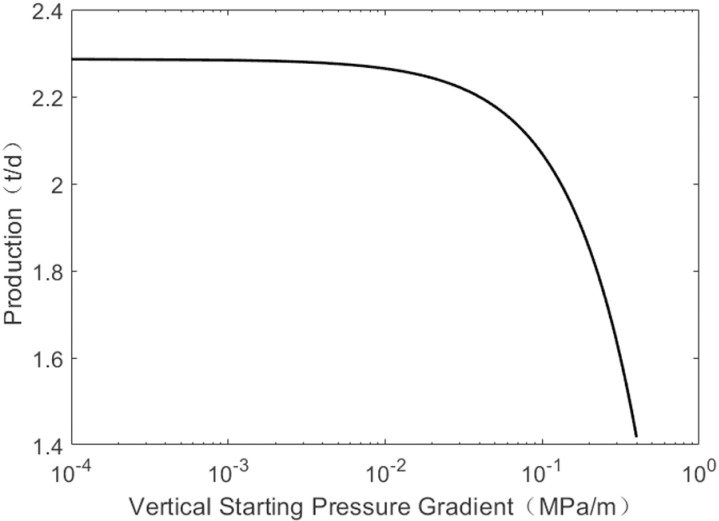
The change of pressure under vertical of start-up pressure gradient.

As shown in Fig 7, production gradually decreases with an increase in the horizontal starting pressure gradient. However, when the horizontal starting pressure is low, production remains basically unchanged. When the horizontal starting pressure gradient exceeds 0.09 MPa/m, oil well production declines sharply. The influence law of the vertical starting pressure gradient on production is basically similar. As shown in Fig 8, when the vertical starting pressure gradient is less than 0.06 MPa/m, the change in production is minimal, but when it exceeds 0.06 MPa/m, production drops sharply. The decline in daily production of oil wells can be attributed to the relationship between the starting pressure gradient and reservoir permeability: the lower the reservoir permeability, the higher the starting pressure gradient. Since the vertical permeability is less than the horizontal permeability, the vertical starting pressure gradient is greater than the horizontal starting pressure gradient. Consequently, oil well production is influenced by both vertical and horizontal starting pressure gradients, but the horizontal starting pressure plays a major role in the production.

## 4. Oil field application

**Fig 9:**
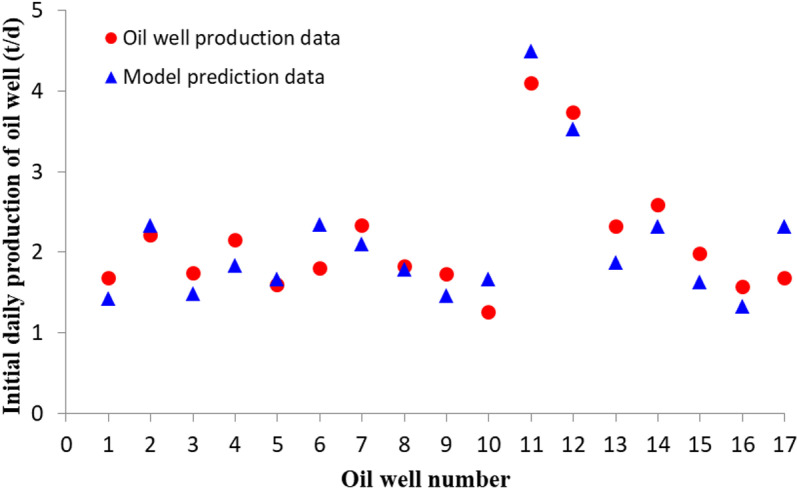
Comparison between initial production capacity predicted by the model and actual production results.

The Chang-6 oil reservoir in the eastern part of the Ordos Basin is a typical shallow oil and gas reservoir. Due to the influence of bedding development, horizontal fractures are generated during fracturing. Therefore, 17 typical fracturing oil wells were selected in this area, with a permeability of 0.6–3.2 × 10^-3^ µm^2^, an original formation pressure of 4.2–6.5 MPa, an oil layer thickness of 3–8 m, a crude oil viscosity of 2.8–4.3 mPa.s, a fracturing half fracture length of 40–100 m, and a starting pressure gradient of 0.002–0.06 MPa/m. The comparison between the production results of the oil wells and the predicted results of the model is shown in Fig 9. It can be seen that the initial production capacity of the oil wells predicted by the model has a high fitting accuracy with the actual production results. The model has a good prediction effect on the initial production capacity of the oil wells and provides a new method for on-site production capacity construction in the oil fields.

## 5. Conclusions

(1) The equivalent seepage resistance method is used to divide the seepage field of fractured horizontal fractures in vertical wells into external field and internal field. Based on the principle of hydropower similarity, the external and internal resistances are combined in series, leading to the establishment of a productivity prediction model of fractured horizontal fractures in vertical wells.
(2) This model can simulate the influence of formation pressure, major and minor axes of fractures, horizontal and vertical permeability of reservoirs, and horizontal and vertical starting pressure gradients on the productivity of fractured horizontal fractures in vertical wells. The simulation results indicate that horizontal permeability is the main factor influencing the daily production of oil wells. The bigger the minor and major axes of elliptical horizontal fracture, the higher the daily production of oil well, with the influence of minor axis length on production being slightly greater than that of major axis. There are critical values for the influence of the starting pressure gradient on production. The critical value of the horizontal starting pressure gradient is 0.09 MPa/m, while that of the vertical starting pressure gradient is 0.06 MPa/m. When the starting pressure gradient is below these critical values, it has a negligible effect on production; however, when it exceeds these values, oil well production drops rapidly.
(3) The model has a high fitting accuracy between the predicted production capacity and the actual production results of the oilfield. The new production model as well as the analysis results of the influencing factors provide valuable guidance for oilfield development design and economic evaluation. Additionally, the productivity model of fractured horizontal fractures in vertical wells offers a certain theoretical basis for the development of low permeability oil reservoirs.
